# The Influence of pH-Driven Interaction Between Soy Protein Isolate and Soy Isoflavones on the Structural and Functional Properties of Their Complexes

**DOI:** 10.3390/foods14223951

**Published:** 2025-11-18

**Authors:** Jing Yang, Yanling Lu, Yanmei Deng, Jiaojiao Yang, Lei Guo, Fangyu Fan

**Affiliations:** 1 College of Biological and Food Engineering, Southwest Forestry University, Kunming 650224, China; yangjing02822@163.com (J.Y.); luyanling20220222@163.com (Y.L.); 14736649465@163.com (Y.D.); aw11p6@163.com (J.Y.); 2 Forest Resources Exploitation and Utilization Engineering Research Center for Grand Health of Yunnan Provincial Universities, Southwest Forestry University, Kunming 650224, China; 3 Key Laboratory of Forest Disaster Warning and Control of Yunnan Province, Southwest Forestry University, Kunming 650224, China

**Keywords:** soy protein isolate, soy isoflavones, pH-driven, protein structure, functional properties

## Abstract

Soy protein isolate (SPI)–soy isoflavone (SI) complexes were prepared via a pH-driven method at varying SI concentrations (0–7.0 mg/mL) to study their interactions and to analyze the structural and functional characteristics of the complexes. The findings showed that the SPI-SI complexes’ particle size first reduced and then rose with higher SI concentration. Fourier transform infrared spectroscopy revealed a reduction in β-sheet content (35.72%), indicating a limited structural rearrangement toward increased conformational flexibility. Consistently, fluorescence spectroscopy revealed that the fluorescence intensity of SPI diminished with the addition of SI, suggesting hydrogen-bond-mediated interactions. At 3.0 mg/mL SI, the complexes exhibited optimal physicochemical properties, with the most negative zeta potential (−29.78 mV), a solubility increase from 41.90% to 64.70%, and an elevated denaturation temperature from 86.77 °C to 88.79 °C, as well as 1.25-fold higher emulsifying capacity, and 1.86-fold greater emulsion stability; collectively establishing 3.0 mg/mL SI as the concentration where functional advantages are maximized. Overall, these findings demonstrate that pH-induced, non-covalent SPI-SI complexation effectively modulates protein conformation and interfacial properties, providing a green and effective approach for enhancing plant protein functionality in food systems.

## 1. Introduction

Soy isoflavones (SI) are flavonoid-type secondary metabolites naturally present in soybean [1]. Their phenolic hydroxyl groups and estrogen-like structures endow them with diverse biological activities, including antioxidant capacity, endocrine-modulating effects, bone protection, and cardioprotective benefits [2]. Because of these bioactivities, SI has gained notable attention in the industries of food, pharmaceuticals, and cosmetics. However, their physicochemical instability under heat, oxygen, and light exposure, combined with low bioavailability, limits their direct incorporation into functional food products [3]. To overcome these limitations, forming protein–polyphenol complexes has been considered a successful strategy to integrate the nutritional value of proteins with the bioactivity of polyphenols. In this context, exploiting SI to modify plant proteins represents a promising strategy to enhance protein functionality and expand their applications.

Soy protein isolate (SPI) is extensively used as a protein from plant origins, offering a balanced amino acid composition, good nutritional value, and large-scale availability [4]. Nevertheless, the compact conformation and poor solubility of SPI result in weak emulsification and limited interfacial activity, restricting its application in functional and high-value-added food formulations [5]. Recent research indicates that non-covalent forces like hydrogen bonding, hydrophobic interactions, and π–π stacking, polyphenols interacting with proteins can adjust their conformation and enhance their functional qualities [6]. For instance, interactions between plant proteins and cinnamic acid, catechin, or rosmarinic acid were found to increase solubility, emulsifying ability, and foaming stability [7]. However, existing research has predominantly focused on phenolic acids and tea polyphenols, leaving the interactions between soy protein and its endogenous isoflavones relatively unexplored [8,9,10]. Yet, conventional complexation methods usually rely on high-shear homogenization or organic solvents, making them environmentally burdensome and difficult to scale up.

The pH-driven method has recently emerged as a green, controllable, and solvent-free strategy for constructing protein–polyphenol complexes. This approach involves reversible conformational transitions of proteins during alkaline unfolding and then refolding during neutralization to form stable complexes through non-covalent interactions. Under alkaline conditions, protein structures partially unfold owing to the reduction in hydrogen bond strength and the rise in electrostatic repulsion, thereby exposing buried hydrophobic residues. At the same time, polyphenols deprotonate and dissolve more readily, which facilitates interaction with the unfolded protein chains [11]. During the neutralization process, proteins refold into compact conformations, and stable non-covalent complexes are formed due to hydrogen bonding and hydrophobic interactions [12]. This mild, solvent-free approach has been successfully employed to enhance the stability and dispersibility of bioactive compounds such as curcumin and catechins, thereby enhancing their functional performance [13]. Despite these advances, the interaction mechanism and functional consequences of SPI-SI complexes prepared using the pH-driven method remain insufficiently understood. Notably, the influence of SI concentration on protein binding, conformational dynamics, and macroscopic functionality remains poorly understood. Clarifying these mechanisms is of great significance for developing controllable and sustainable strategies for plant protein modification.

Therefore, this study aimed to elucidate how the concentration of SI modulates the formation, structural characteristics, and interfacial functionality of SPI-SI complexes prepared through a pH-driven assembly process without organic solvents. Specifically, we investigated the effects of SI concentration on particle size, zeta potential, conformational transitions, and intermolecular interactions of the complexes, as well as how these structural changes relate to emulsifying activity, emulsion stability, surface hydrophobicity (H_0_), and thermal stability. The findings are expected to provide a mechanistic basis for employing SPI-SI complexes as a process-friendly, clean-label, and sustainable approach to tune the functional performance of plant proteins in plant-based emulsions and related food systems.

## 2. Materials and Methods

### 2.1. Materials

The supplier of soybean meal was Tianjin Zhongchuliang Zhenjiang Grain and Oil Co., Ltd. (Tianjin, China). Genistein (purity > 98%) was obtained from Shanghai Source Leaf Biological Technology Co., Ltd. (Shanghai, China). Soybean oil suitable for food use was obtained from Yihai Kerry Food Industry Co., Ltd. (Kunming, China). Chemicals of analytical grade were used throughout this study.

### 2.2. Preparation of SPI

Soybean meal underwent defatting followed by a sequential protocol: lipid removal, alkaline extraction, and isoelectric precipitation. The resulting SPI contained 91.70% crude protein (dry basis) as established using the Kjeldahl process [14].

### 2.3. Preparation of SI

Defatted soybean meal (240 g) was separated using 4800 mL of 70% (*v*/*v*) ethanol under ultrasonication (25 kHz, 40 min, 60 °C). Following extraction, the blend was permitted to return to room temperature before being centrifuged (6793× *g*, 10 min). The supernatant fluid was taken, purified using AB-8 macroporous resin, and eluted using 80% (*v*/*v*) ethanol at 1 mL/min. The eluate was concentrated under reduced pressure (40 °C, 0.1 mbar, 15 min) using a rotary evaporator (N-1100D, Ankeyq Instrument Co., Ltd., Shanghai, China), and freeze-drying of the remaining solution was performed at −60 °C under a pressure of 0.1 mbar 48 h using a Scientz-10ND freeze dryer (Ningbo Scientz Biotechnology Co., Ltd., Ningbo, China) to obtain SI [15].

A genistein standard (0.21 g/L stock) was serially diluted to concentrations of 2.10–10.60 mg/L. Measurements of absorbance were carried out at 265 nm. A UV-2600 spectrophotometer (Shimadzu Corporation, Kyoto, Japan) was employed, and 70% (*v*/*v*) ethanol was used as the blank reference [16]. The calibration curve was *y* = 0.3142 *x* − 0.0385 (R^2^ = 0.9997). To quantify the SI content, an amount of 1 g of the sample was dissolved in 25 mL of 70% (*v*/*v*) ethanol, followed by filtration. Further dilution of the filtration was performed to achieve the desired absorbance within the calibration curve range. Using the corresponding regression equation, the concentration and corresponding purity (65.70%) of SI were determined.

### 2.4. Preparation of SPI-SI Complexes

**Figure 1:**
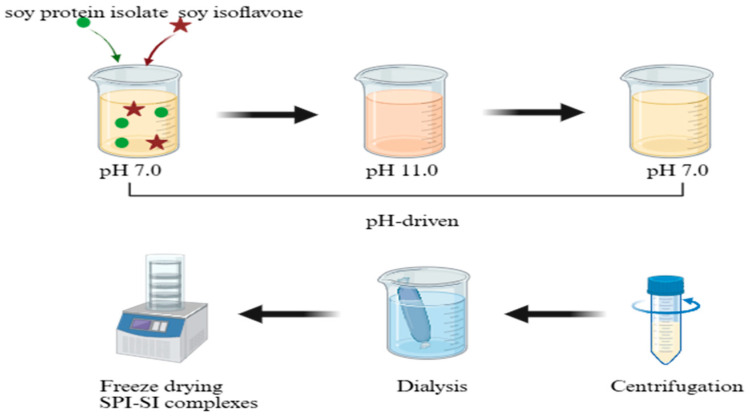
Preparation process of SPI-SI complexes by pH-driven method.

As shown in Figure 1, a 1% (*w*/*v*) SPI powder solution was prepared by dispersing it in deionized water and stirring magnetically (150 rpm, 30 min), after which the pH was adjusted to 11.0 using 2 mol/L NaOH. The SPI solution was stirred magnetically (150 rpm, 30 min) after which the SI ethanolic solution was added with SI concentrations of 0, 1.0, 3.0, 5.0, and 7.0 mg/mL. The mixed solution was magnetically stirred (150 rpm, 2 h). After filtration, ethanol in the solution was evaporated under reduced pressure (40 °C, 0.1 mbar) using a rotary evaporator. The solution pH was adjusted to 7.0 with 2 mol/L HCl, and ultrasonic treatment was applied (40 kHz, 40 min, 25 °C). The blend was subjected to centrifugation (6793× *g*, 10 min), followed by 48 h of dialysis. The mixtures were subsequently lyophilized at −60 °C and 0.1 mbar for 48 h using a freeze-dryer to yield SPI-SI complexes, labeled as SPI, SPI-SI1, SPI-SI3, SPI-SI5, and SPI-SI7 [17]. The pH adjustment procedure was identical for all samples, ensuring consistent ionic strength conditions across treatments during complex formation.

### 2.5. Characterization of the SPI-SI Complexes

#### 2.5.1. Particle Size, Polydispersity Index, and Zeta Potential Analysis

The particle size, polydispersity index (PDI), and zeta potential of the samples were evaluated using a Zetasizer Nano ZS90 (Malvern Panalytical Ltd., Malvern, UK). Deionized water, having a refractive index of 1.33, served as the medium, while the solute concentration was established at 1.0 mg/mL. Data collection occurred at 25 °C.

#### 2.5.2. Fourier Transform Infrared Spectroscopy Analysis

The Fourier-transform infrared (FTIR) spectra were recorded using an FTIR-650 spectrometer (Tianjin Gangdong Science & Technology Development Co., Ltd., Tianjin, China). Each 1 mg sample was finely mixed with 100 mg of pre-dried KBr and pressed into a transparent pellet. Spectral measurements were carried out at 25 °C across 400–4000 cm^−1^ using a 4 cm^−1^ resolution, and 16 scans were averaged to obtain a better signal-to-noise ratio. The amide I band (1600–1700 cm^−1^) was deconvoluted and curve-fitted using PeakFit v4.12 to evaluate variations in the secondary structure [18].

#### 2.5.3. Fluorescence Spectroscopy Analysis

The fluorescence of the samples was analyzed using a SpectraMax Plus384 microplate reader (Molecular Devices, LLC, San Jose, CA, USA). The sample solutions (1.0 mg/mL) were prepared in phosphate-buffered saline (0.10 mol/L, pH 7.0). The excitation wavelength was 280 nm, and emission spectra were measured from 300 to 500 nm, with the excitation and emission slit widths maintained at 5 nm [19].

#### 2.5.4. Ultraviolet Spectroscopy Analysis

The UV-2600 spectrophotometer was employed to capture ultraviolet spectra. The samples were dissolved in PBS (0.10 mol/L, pH 7.0) to yield 1.0 mg/mL solutions, which were analyzed by means of scanning from 200 to 400 nm at 25 °C using a 5 nm slit width [20].

#### 2.5.5. Molecular Docking Analysis

Using OpenBabel (version 3.1.1), the three primary aglycones of SI (daidzein, glycitein, and genistein) were obtained from PubChem in SDF format and transformed into PDB format. From the RCSB Protein Data Bank (RCSB PDB: Homepage), crystal structures for β-conglycinin (PDB: 1UIJ) and glycinin (PDB: 1FZX) were acquired. With PyMOL (version 3.1.0), hydrogen atoms were added manually, and every water molecule was removed. Docking input files and grid parameters were prepared using AutoDockTools (version 1.5.7). AutoDock Vina (version 1.2.0) was used to carry out molecular docking in blind docking mode, allowing the search across the entire protein surface. Default settings were used for all Vina docking parameters. The binding modes and molecular interactions of the resulting complexes were visualized and analyzed with PyMOL.

#### 2.5.6. Atomic Force Microscopy Observation Analysis

Atomic force microscopy (AFM) was employed to analyze the surface topography of the samples. The dispersion was prepared by diluting the samples in deionized water to a final concentration of 100 µg/mL. Subsequently, a droplet of the suspension was cast onto freshly cleaved mica and dried under ambient temperature and humidity prior to imaging. AFM images were recorded in tapping mode using a Dimension Icon instrument (Bruker Nano GmbH, Karlsruhe, Germany) with a scanning area of 3 µm × 3 µm.

#### 2.5.7. Thermal Stability Analysis

Thermal stability of SPI-SI complexes was analyzed using a 3500 Sirius differential scanning calorimeter (Netzsch-Gerätebau GmbH, Selb, Germany). Each dried sample (0.2 mg) was encapsulated in an aluminum pan, with an empty pan used as a reference. The thermal scan was carried out from 25 to 200 °C at a rate of 10 °C/min with a nitrogen purge rate of 20 mL/min. The denaturation temperature (T_d_) was determined from the endothermic peak of the thermogram [21].

#### 2.5.8. Solubility Analysis

Sample solutions were prepared in 0.10 mol/L PBS (pH 7.0) at a concentration of 0.1 g/mL, followed by centrifugation (6793× *g*, 10 min). The Coomassie Brilliant Blue method was used to evaluate the protein content in the supernatant. Determining the solubility of proteins was performed in accordance with Equation (1) [22].

(1) $$\mathrm{Solubility}\;(\%)=\frac{C\times V}{m\times 1000}\times 100$$

where *C* represents the protein concentration in the supernatant (mg/mL), *V* denotes the volume (mL), and *m* stands for the protein mass (g).

#### 2.5.9. Surface Hydrophobicity Analysis

Solutions with concentrations of 0.1, 0.2, 0.5, 1.0, 2.0, and 5.0 mg/mL were obtained by dissolving the samples in 0.10 mol/L PBS (7.0). Subsequently, the sample solution was mixed with 40 μL of a 1-anilino-8-naphthalenesulfonate (ANS) solution at a concentration of 0.008 mol/L and permitted to stand for 15 min. The SpectraMax Plus384 microplate reader was used to record fluorescence, applying excitation and emission wavelengths of 390 nm and 470 nm, with 5 nm slit settings. A standard curve was constructed by plotting sample concentration (*x*-axis) against fluorescence intensity (*y*-axis); the Surface hydrophobicity (H_0_) index was determined using the slope of the linear region, which reflects the extent of hydrophobic groups exposed on the protein surface [23].

#### 2.5.10. Surface Free Sulfhydryl Content Analysis

The sample solution (1.0 mg/mL) was blended with Tris-Glycine buffer solution (0.086 mol/L Tris, 0.090 mol/L Glycine, 0.004 mol/L Ethylenediaminetetraacetic acid, pH 8.0) at a 1:4 (sample–buffer) ratio. Then, a volume of 20 μL of Ellman’s reagent, with a concentration of 4.0 mg/mL (396 mg of 5,5′-Dithiobis-(2-nitrobenzoic acid) in 100 mL of 0.1 mol/L Tris-Glycine buffer), was then added. After vortex mixing for 30 s, the samples underwent a 30 min incubation at 25 °C in the dark, with reagent blanks (buffer only). The UV-2600 spectrophotometer was employed to measure absorbance at 412 nm, and the surface free sulfhydryl (-SH) content was determined as per the procedure in Equation (2) [24].

(2) $$\mathrm{Surface}\;\mathrm{free}\text{-}\mathrm{SH}\;\mathrm{groups}\;(\mu L/g)=\frac{(75.53\times A_{412})\times n}{C}$$

where *A*_412_ represents the sample’s absorbance at 412 nm, *C* indicates the concentration of the sample (1.0 mg/mL), and *n* refers to the dilution factor (*n* = 100).

#### 2.5.11. Emulsifying Activity Index and Emulsifying Stability Index Analysis

A volume of 15 mL of the sample solution at a concentration of 1.0 mg/mL was combined with 5 mL of soybean oil in a test tube. This mixture was combined and subjected to an FJ200-SH high-speed shear (Shanghai Luce Industrial Co., Ltd., Shanghai, China) (10,000 rpm, 1 min). Then, a volume of 50 μL of emulsion was extracted from the bottom of the test tube at both 0 and 10 min and diluted 100-fold using a 1.0 mg/mL sodium dodecyl sulfate (SDS) solution. Absorbance at 500 nm was determined using a UV-2600 spectrophotometer, and the calculations for emulsifying activity index (EAI) and emulsifying stability index (ESI) were based on Equations (3) and (4) [25].

(3) $${\mathrm{EAI}\;(m}^{2}/g)=\frac{2\times 2.303\times A_{0}\times 100}{c\times (1-\varphi )\times 10000}$$

(4) $$\mathrm{ESI}\;(\min)=\frac{A_{10}}{A_{0}}\times 100$$

where *A*_0_ and *A*_10_ represent the absorbance of the sample solution at 0 and 10 min, respectively; *c* is the concentration of the sample (1.0 mg/mL); *φ* represents the oil phase’s volume fraction (25%, *v*/*v*).

#### 2.5.12. Water Holding Capacity and Oil Binding Capacity Analysis

Each 0.5 g sample was mixed thoroughly with 15 mL of deionized water or soybean oil in a 25 mL centrifuge tube. Following pH adjustment to 7.0, the prepared suspensions were incubated in a water bath at 60 °C for 30 min. The samples were then centrifuged at 10,615× *g* for 5 min, and the resulting supernatants were decanted. The water holding capacity and oil binding capacity (WHC and OBC) were calculated based on Equations (5) and (6) [26].

(5) $$\mathrm{WHC}\;(\%)=\frac{m_{2}-m_{1}-0.5}{0.5}$$

(6) $$\mathrm{OBC}\;(\%)=\frac{m_{2}-m_{1}-0.5}{0.5}$$

where *m*_1_ represents the mass of the centrifuge tube (g), and *m*_2_ is the combined mass of the centrifuge tube and the precipitation following the centrifugation process (g).

### 2.6. Statistical Analysis

Each experiment was conducted in triplicate using independently prepared samples (*n* ≥ 3). Quantitative data are presented as the mean ± standard deviation (SD) from these independent replicates. For spectroscopic measurements, a representative spectrum is shown. To analyze variance (ANOVA), SPSS 24.0 was employed, and Duncan’s test (*p* < 0.05) was employed to evaluate the significance of the data differences.

## 3. Results and Discussion

### 3.1. Particle Size and Polydispersity Index

**Figure 2:**
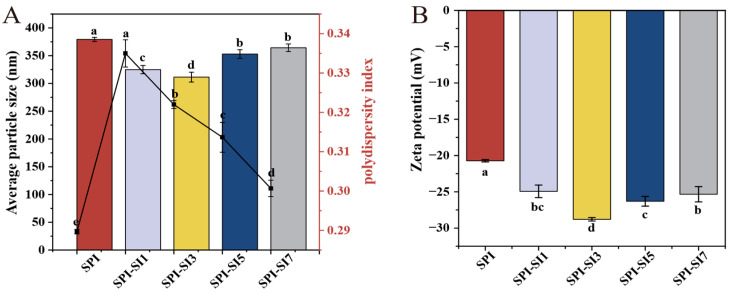
Average particle size (bars, left *Y*-axis) and polydispersity index (curve, right *Y*-axis) (**A**), and zeta potential (**B**) of SPI-SI complexes. Data are presented as the mean ± standard deviation (SD, *n* = 3). Distinct lowercase letters indicate statistically significant differences between samples (*p* < 0.05).

Figure 2A illustrates that the average size of the particles in SPI measures 379.32 nm. Upon complexation with increasing SI concentrations, the SPI-SI complexes’ average particle size initially decreased and then increased. For SPI-SI1 and SPI-SI3, the particle sizes were notably reduced in comparison to SPI (*p* < 0.05), reaching a minimum value of 311.42 nm in SPI-SI3. This decrease might be due to conformational changes in SPI caused by SI, compacting the protein chains and reducing steric hindrance [27]. Therefore, a decrease in particle size was initially observed. However, a further elevation in SI concentration (SPI-SI5 and SPI-SI7) caused the particle size to increase. The observed particle aggregation may result from enhanced hydrophobic interactions between SPI and SI, which facilitate the formation of larger assemblies. Correspondingly, at low SI concentration (SPI-SI1), the PDI was significantly higher than the SPI (0.29) (*p* < 0.05), implying insufficient interaction to narrow the particle size distribution. As SI concentrations increased (from SPI-SI3 to SPI-SI7), the PDI decreased, indicating a tendency toward narrower size distribution and improved dispersion uniformity. When the PDI approached 0.3, physical stability remained favorable [28]. Therefore, although PDI values remained around the acceptable threshold and did not fall below that of SPI, the distribution of particle sizes became narrower with increasing SI concentration. Similar fluctuations in particle size after binding dietary flavonoids to soybean protein have been described by Jia et al. [29], which supports the current findings. Overall, the SPI-SI system demonstrated optimal dispersibility and stability at the SI concentration (SPI-SI3), while excessive SI promoted aggregation, thereby limiting potential gains in functional performance.

### 3.2. Zeta Potential

The zeta potential reflects the electrostatic properties of the particle surface, and a greater absolute value typically indicates enhanced dispersion stability [30]. Figure 2B illustrates that all samples exhibited negative zeta potentials, with SPI at −20.69 mV. Upon SI addition, the zeta potential became more negative (absolute value increased), reaching a maximum absolute value of −28.79 mV for SPI-SI3 (*p* < 0.05). The increase in negative charge is probably associated with the adsorption of SI molecules on the particle interface, which may alter the surface configuration and render more negatively charged sites accessible [31]. Therefore, electrostatic repulsion and dispersion stability are strengthened at SPI-SI3. Nevertheless, as the SI concentration (SPI-SI5 and SPI-SI7) increased, the absolute value of the zeta potential consistently diminished. This decrease likely results from aggregation and charge screening at higher SI concentrations, which can limit the effective exposure of surface charges and reduce electrostatic repulsion between particles. Therefore, dispersion stability likely declined at higher SI concentrations. Similar behavior has been reported for other protein–polyphenol complexes, where excessive polyphenol induces bridging and aggregation, leading to diminished zeta potential [32]. Collectively, these observations indicate that moderate SI supplementation enhances the negative surface charge and dispersion stability of SPI-SI complexes, whereas excessive SI addition exerts the opposite effect. Conversely, excessively high SI concentrations result in a reduction of zeta potential, indicating compromised dispersion stability. Therefore, the stabilization effect of SI is most prominent at SPI-SI3, while further increases in SI concentrations were not further investigated in this study due to their potential negative impact on system stability and functionality.

### 3.3. Fourier Transform Infrared Spectroscopy

**Figure 3:**
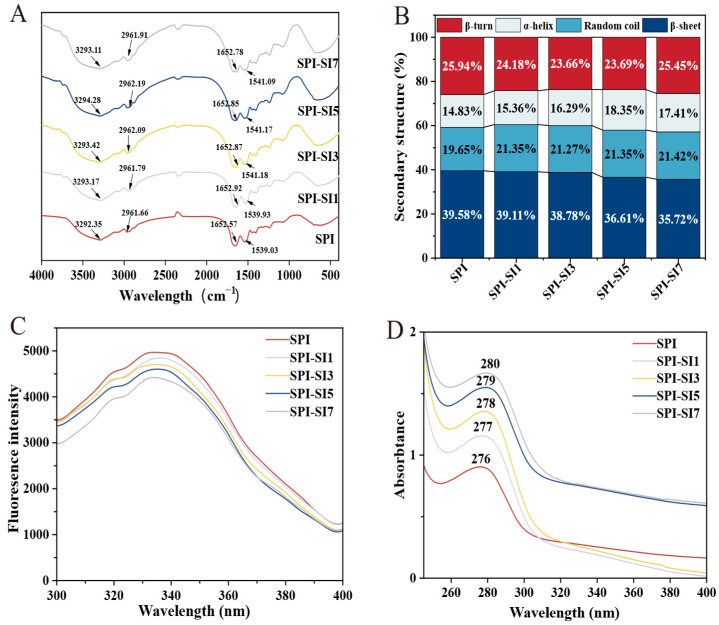
Fourier transform infrared spectra (FTIR) (**A**), secondary structure content (**B**), fluorescence spectra (**C**), and ultraviolet spectra (**D**) of SPI-SI complexes. Data for panel (**B**) are displayed as mean ± SD (*n* = 3), and panels (**A**,**C**,**D**) illustrate representative spectra from triplicate experiments.

The interaction mechanisms between SPI and SI are depicted in Figure 3A using FTIR spectra. At 3292 cm^−1^, 2961 cm^−1^, 1652 cm^−1^, and 1530 cm^−1^, SPI’s characteristic peaks were observed. Compared with SPI, the SPI-SI complexes showed a slight shift in the range of 3292 to 3293 cm^−1^ for the O-H/N-H stretching band, which is attributed to changes in hydrogen bonding interactions, suggesting that SPI and SI likely form hydrogen bonds. The band near 2960 cm^−1^ corresponding to C-H stretching of CH_2_/CH_3_ also showed a minor change upon SI addition, reflecting adjustments in the hydrophobic microenvironment as SPI interacts with SI [33]. The absorption peaks were identified at about 1649 cm^−1^ and 1539 cm^−1^, resulting from interactions between SPI and SI, which cause changes in the vibrational modes of C=O and N-H, consistent with modifications in polarity, charge distribution, and intermolecular interactions that can impact SPI-SI complexes’ solubility and stability [34]. Together, these subtle band shifts support non-covalent SPI-SI interactions.

The FTIR findings suggest a modification in the amide I band of SPI-SI complexes, showing an alteration in the secondary structure of these complexes in relative to SPI. As shown in Figure 3B, increasing SI concentrations (from SPI-SI1 to SPI-SI7) led to a gradual decrease in β-sheet content (from approximately 39.58% to 35.72%) and a slow decrease in β-turn, accompanied by small increases in α-helix and random coil contents (from about 14.83% to 17.41% and from 19.65% to 21.42%, respectively). These are modest changes and may be close to the uncertainty of curve fitting; nevertheless, they suggest a slight redistribution of secondary structure toward higher α-helix/random coil. Overall, SI appears to induce limited chain rearrangement and slightly increased conformational flexibility via hydrogen bonding and hydrophobic interactions, which may contribute to modified solubility and interfacial behavior of the complexes. This redistribution is consistent with increased chain flexibility, as reported for other protein–ligand systems [35]. These findings indicate that increasing SI concentration is associated with a slight redistribution from β-sheet/β-turn toward α-helix/random coil, potentially stabilizing SPI-SI complexes [36]. Li et al. [37] demonstrated that the secondary structure transitions observed in protein–polyphenol systems underscore the regulatory effect of polyphenols on protein conformation. Overall, the findings indicate that elevated SI concentrations modulate the secondary conformation of SPI, consequently affecting its structural and functional behavior.

### 3.4. Fluorescence Spectra

Fluorescence measurements of tryptophan (Trp) and tyrosine (Tyr) residues–both mainly excited at 280 nm, with phenylalanine being less excited–are particularly sensitive to the local environment [38]. Thus, the fluorescence serves as a direct probe for monitoring conformational shifts in protein–polyphenol systems [39]. Figure 3C illustrates the emission spectra of SPI-SI complexes and SPI. At a constant SPI concentration, there was a notable reduction in fluorescence intensity (*p* < 0.05) with increasing SI concentrations, from 4816 (SPI-SI1) to 4416 (SPI-SI7), compared with 4968 for SPI. This decrease indicates that SI binding alters SPI conformation, leading to reduced fluorescence intensity. Comparable reductions in fluorescence intensity observed in different protein–polyphenol systems indicate alterations in the environment around aromatic residues [40]. The emission maximum of SPI was 333 nm, which red-shifted to 336 nm at SPI-SI3, indicating that SI interaction rendered the Trp microenvironment more polar [41]. This behavior resulted in a slight red shift. However, with further SI concentrations (from SPI-SI3 to SPI-SI7), the emission maximum blue-shifted back toward 333 nm, suggesting progressive burial of Trp residues in hydrophobic domains due to increased SI occupancy and protein compaction, which reflects enhanced hydrophobic packing at higher SI concentrations [42]. Therefore, the blue shift was observed. According to Yan et al. [43], upon the formation of SPI–epigallocatechin gallate conjugates, the fluorescence signal weakened and the emission maximum exhibited a noticeable blue shift.

### 3.5. Ultraviolet Spectra

Ultraviolet spectra provide a non-destructive approach to investigate protein–polyphenol interactions by tracking the absorption characteristics of aromatic residues, which are sensitive to changes in protein conformation and their surrounding microenvironment [44]. Figure 3D presents the ultraviolet spectra of SPI-SI complexes. SPI exhibited an absorption peak at 276 nm, arising from π–π transitions of aromatic residues. Upon increasing the SI concentrations (from SPI-SI1 to SPI-SI7), there was an increase in the absorption intensity of the SPI-SI complexes, with a red shift from 277 to 280 nm. The enhanced absorption and slight red shift suggest that SI binding may increase chromophore exposure and perturb electronic transitions, mainly by means of π–π stacking and hydrogen bonding interactions with aromatic residues, as well as increased local polarity. These interactions collectively influence the conformation of SPI and alter the immediate chromophore environment [45]. Comparable spectral variations were observed by You et al. [46], who demonstrated that complexes between SPI and gallic acid esters also exhibited red shifts and increased absorption. Collectively, these findings suggest that SI association modifies the electronic environment of aromatic amino acids and contributes to conformational reorganization of SPI, which helps in forming stable SPI-SI complexes.

### 3.6. Molecular Docking

**Figure 4:**
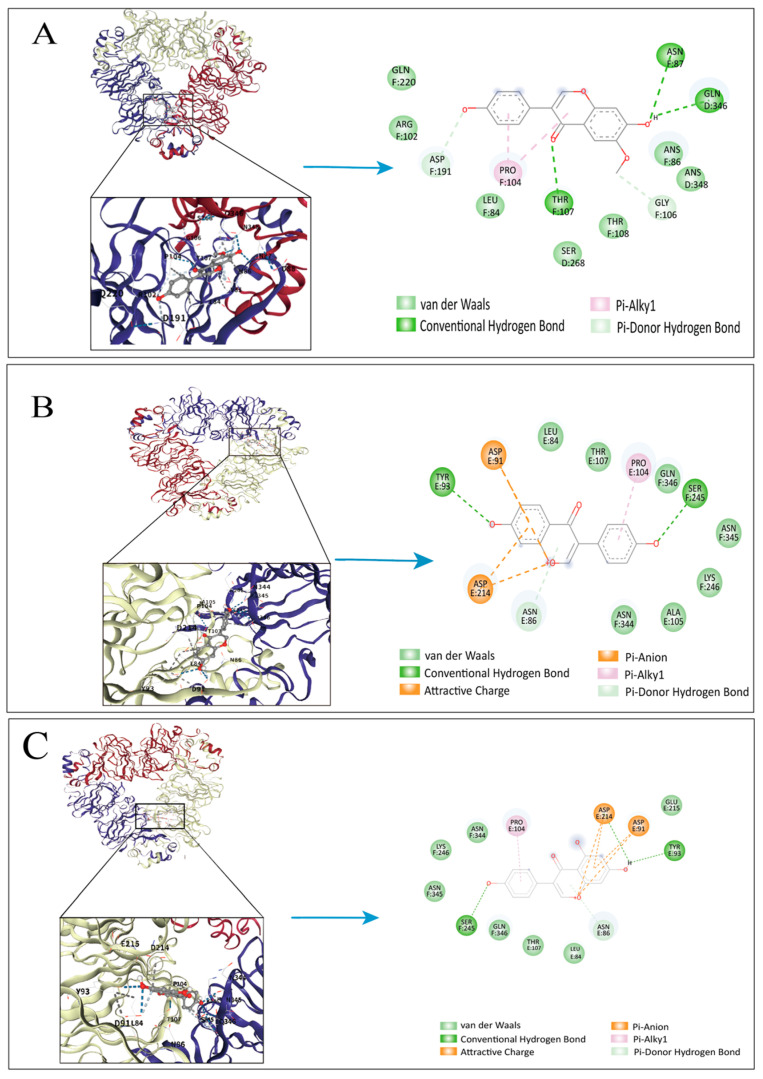

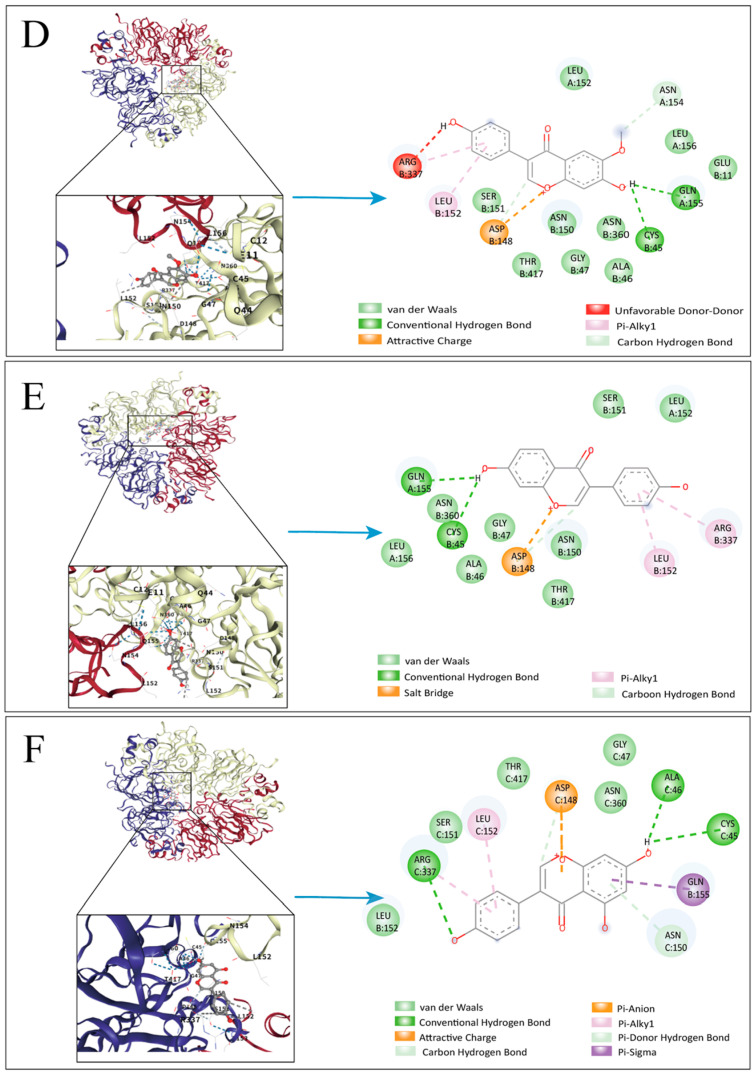
The 3D docking model, 2D interaction schematic interaction between β-conglycinin and Glycitein (**A**), Daidzein (**B**), Genistein (**C**), Glycinin and Glycitein (**D**), Daidzein (**E**), Genistein (**F**), and Autodock Vina indicates the interaction with the minimum energy from left to right. Green dashed lines represent conventional hydrogen bonds; light-green lines indicate van der Waals interactions; mint-green lines represent carbon–hydrogen bonds (weak hydrogen bonds). Pink dashed lines represent π–alkyl interactions; orange lines indicate attractive charge or π–anion interactions; cyan lines represent π–donor hydrogen bonds; purple lines denote π–sigma interactions; and red dashed lines indicate unfavorable donor–donor or acceptor–acceptor interactions. In the 2D diagrams, the labels “O”, “+”, or “−” next to red lines indicate the interacting atoms or charge centers (oxygen, positive, or negative sites, respectively).

A theoretical study was carried out to explore the interacting mechanism between SPI and SI, using molecular docking. This work focused on the two primary subunits, 7S and 11S, together with three bioactive isoflavone aglycones—daidzein, glycitein, and genistein. The docking arrangements that exhibit the strongest binding affinities were chosen for analysis, as illustrated in Figure 4, which displays the three-dimensional binding structures and two-dimensional interaction diagrams of 11S and 7S with the isoflavone molecules, respectively. The docking results revealed that all three isoflavones can be accommodated within the hydrophobic cavities of SPI, where multiple amino acid residues stabilize the complexes using non-covalent interactions [47]. For instance, daidzein forms hydrogen bonds with polar amino acids, including Ser245, Thr107, Asn346, and Glu93, exhibiting favorable bond lengths between 2.8 and 3.2 Å. In the case of 11S, glycitein established stable hydrogen bonds with Glu87, Asn218, and Gly243, while its aromatic ring engaged in π-alkyl interactions with Leu84 and Pro104; electrostatic attraction involving Glu87 further contributed to binding stability [22]. Overall, the principal stabilizing forces were hydrogen bonding and hydrophobic interactions (π–π and π-alkyl stacking), while electrostatic interactions played a minor role. These docking findings are consistent with the spectroscopic analyses and previous reports on protein–polyphenol complexes [48].

### 3.7. Atomic Force Microscopy Observation

**Figure 5:**
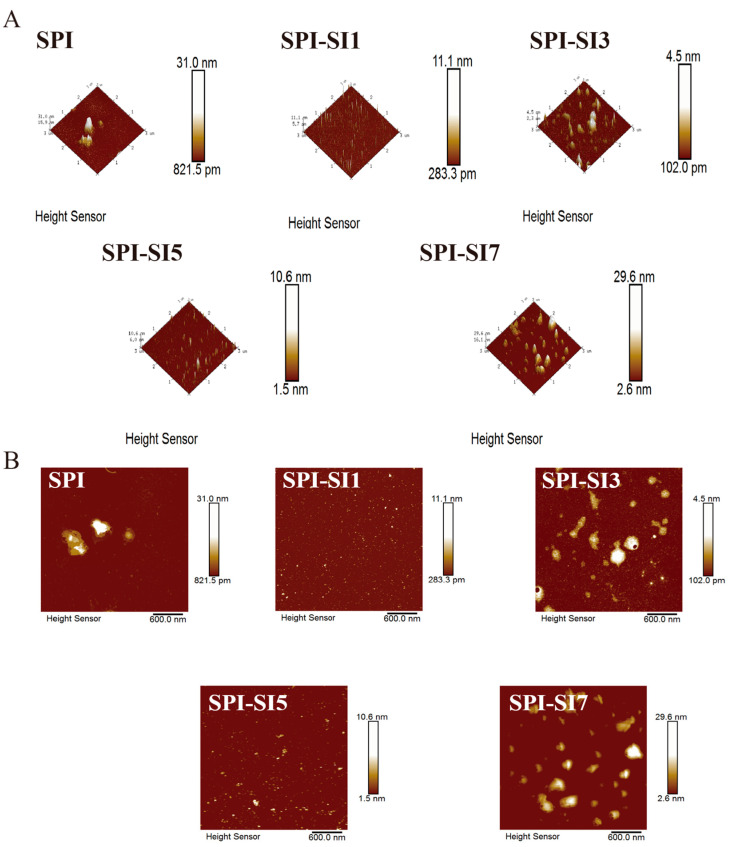
Three-dimensional atomic force microscopy (AFM) images (**A**) and two-dimensional AFM images (**B**) of SPI-SI complexes.

The aggregation state and the surface morphology of SPI and its complexes were characterized using AFM. As illustrated in Figure 5, both two- and three-dimensional images revealed clear morphological differences between SPI and SPI-SI complexes. SPI exhibited large, heterogeneously distributed particles with pronounced height variation and surface roughness (Rq), indicating pronounced aggregation and inhomogeneous surface characteristics. In contrast, SPI-SI complexes generally showed smaller features and improved uniformity, with aggregation markedly reduced. As SI concentrations increased (from SPI-SI1 to SPI-SI3), the surface height and Rq initially decreased, implying that moderate SI levels effectively hindered SPI aggregation and facilitated a more compact surface arrangement. However, further SI addition led to a renewed increase in particle size and Rq, with three-dimensional images revealing more distinct nanoparticle peaks and localized clustering [49]. Overall, AFM confirmed a concentration-dependent morphological evolution: moderate SI reduced particle height/Rq and aggregation, while excessive SI led to re-aggregation and increased roughness. These observations align with the particle size/PDI trends described earlier, reinforcing the consistency of structural and surface characterizations.

### 3.8. Thermal Stability

**Figure 6:**
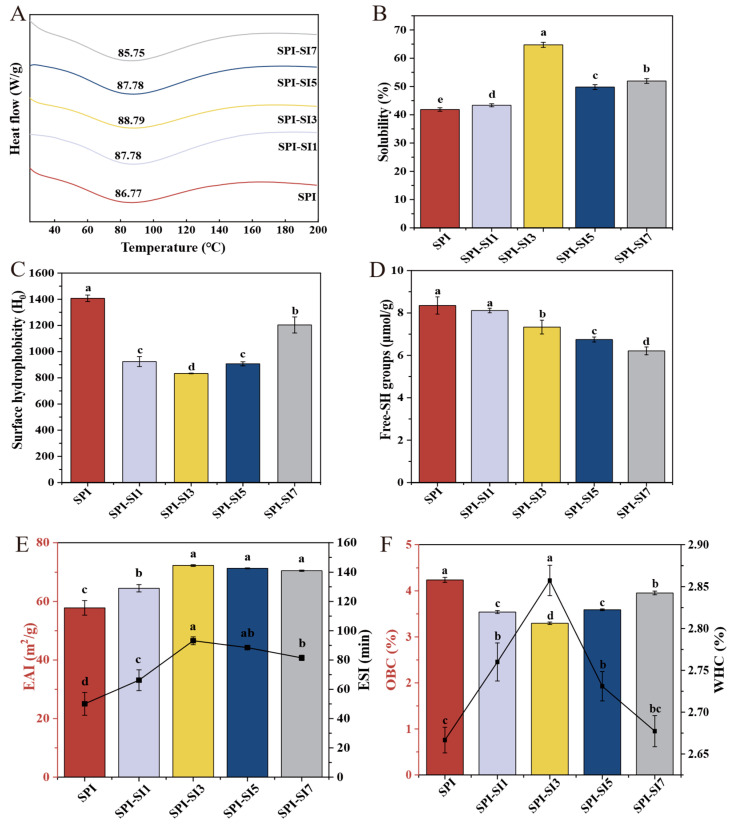
Thermal stability (**A**), solubility (**B**), surface hydrophobicity (H_0_) (**C**), free-SH groups (**D**), emulsification activity index (EAI) (bars, left *Y*-axis) and emulsification stability index (ESI) (curve, right *Y*-axis) (**E**), and water holding capacity (WHC) (curve, right *Y*-axis), and oil binding capacity (OBC) (bars, left *Y*-axis) (**F**) of SPI-SI complexes. Data are presented as the mean ± SD (*n* = 3). Distinct lowercase letters indicate statistically significant differences between samples (*p* < 0.05).

Protein conformational stability can be evaluated by tracking temperature-dependent unfolding transitions during thermal denaturation [21]. Figure 6A illustrates the thermal stability of SPI-SI complexes. T_d_ represents the critical juncture where a protein changes from its natural configuration to a denatured form, with a higher Td generally indicating enhanced thermal stability [50]. The T_d_ of SPI was 86.77 °C. As SI concentration increased (from SPI-SI1 to SPI-SI5), T_d_ rose. This phenomenon is due to hydrogen bonds and hydrophobic interactions that strengthen the protein framework, diminish molecular flexibility, and necessitate increased thermal energy for unfolding. Thus, the T_d_ increased. The T_d_ of SPI-SI7 decreased to 85.75 °C relative to SPI-SI5. This reduction in thermal stability is associated with conformational alterations at excessive SI levels, leading to the formation of large, disordered aggregates. Such aggregation behavior is consistent with the structural loosening and partial unfolding revealed by FTIR and fluorescence analyses, which collectively indicate a transition from an ordered to a less organized state under high SI concentrations. Xu et al. [51] reported that grafting phenolic compounds such as catechins and gallic acid onto myofibrillar protein–dextran conjugates significantly enhanced their thermal stability and antioxidant activity (*p* < 0.05).

### 3.9. Solubility

Figure 6B shows that the solubility of SPI-SI complexes increased significantly compared to SPI (*p* < 0.05). Solubility is defined as the maximum amount of a substance that can dissolve under specific conditions. For proteins, solubility is influenced by molecular conformation, surface charge, and solvent conditions [52]. The solubility of SPI measured at 41.90%, while the solubility of SPI-SI complexes notably rose (*p* < 0.05) with rising SI concentrations, ultimately reaching a maximum of 64.70% for SPI-SI3, which represents a significant increase of 54.40% compared to SPI. This enhancement is likely due to SI-induced conformational adjustments that reduce hydrophobic association and increase exposure of polar groups, thereby increasing net surface charge and hydration [53]. This interpretation is further supported by the concurrent increase in the absolute value of the zeta potential, indicating enhanced electrostatic repulsion and improved dispersion, which together facilitate protein solubility. Therefore, the solubility increased. At excessive SI concentrations, solubility was markedly reduced (*p* < 0.05). This decline could result from the saturation of binding sites and the occurrence of inter-complex aggregation or bridging, as well as partial phase separation, which collectively weakens hydration ability. Moreover, this decline in solubility was directly corroborated by visual observation; the centrifuged solutions of SPI-SI5 and SPI-SI7 exhibited clear turbidity and particulate aggregates, which were absent in the clear solutions of SPI and low concentration complexes. Therefore, the solubility decreased. These observations agree with previous findings that appropriate polyphenol concentrations improve solubility via protein–polyphenol interactions [54,55]. In summary, the solubility is influenced by SI in a manner that depends on its concentration, potentially affecting the functional characteristics and uses of SPI-based food systems.

### 3.10. Surface Hydrophobicity

Alterations in protein structure and the unveiling of hydrophobic regions affect H_0_, which in turn affects solubility, aggregation tendency, and interfacial adsorption behavior [56]. The study employed a fluorescent probe for ANS to examine the SPI-SI complexes, as ANS specifically binds to exposed hydrophobic regions. As shown in Figure 6C, increasing SI concentrations significantly impacted the H_0_ of SPI-SI complexes (*p* < 0.05). The H_0_ of SPI was 1407. After SI was added, H_0_ decreased markedly (*p* < 0.05), with values dropping to 923 for SPI-SI1 and 833 for SPI-SI3. This reduction in H_0_ indicates that moderate SI concentrations mask hydrophobic patches and enhance interfacial hydration (via adsorption of phenolic hydroxyls), thereby reducing the H_0_. Similarly, Wang et al. [57] reported that SPI-tannic acid complexes exhibited decreased H_0_ due to non-covalent interactions, and the carboxyl groups on tannic acid enhanced the surface hydration of SPI. Phenolic compounds can alter the complex structure and net surface charge through binding, thereby affecting the hydrophilic/hydrophobic group on the protein surface [58]. However, with further increases in SI concentration, H_0_ values began to rise again, reaching 907 for SPI-SI5 and 1203 for SPI-SI7. This increase can be attributed to excessive SI binding, which may disrupt the compact structure of SPI and expose previously buried hydrophobic residues and/or promote association, thereby increasing the H_0_ [59]. In the same way, Hao et al. [60] prepared complexes of pea protein isolate with phenolics (e.g., chlorogenic acid, resveratrol) that significantly reduced H_0_ (*p* < 0.05), a trend consistent with the present study. Collectively, these findings demonstrate that SI modulates the H_0_ of SPI in a concentration-dependent manner, which could have profound effects on the stability and functionality of SPI-based food systems.

### 3.11. Surface Free Sulfhydryl

The conformational stability of proteins is closely linked to the presence of surface free-SH groups, which primarily originate from cysteine residues. These reactive moieties possess strong reducing capabilities, enabling participation in diverse biochemical processes [61]. As depicted in Figure 6D, the surface free-SH content of SPI was 8.35 μmol/g. Upon SI incorporation, the SPI-SI complexes exhibited a significant reduction (*p* < 0.05). This is likely related to SI-induced conformational rearrangements and partial aggregation. As SI concentration increased, reactive side chains (e.g., -SH) became progressively more occluded within protein aggregates or underwent further modification, thereby lowering the detectable -SH content [60]. In addition, phenolic compounds may oxidize to form quinones when exposed to alkaline and oxidative environments. These quinones readily engage with the sulfhydryl or amino groups present in SPI, leading to the creation of C-S/C-N linkages. Consequently, this results in a reduction of the free-SH content within SPI-SI complexes [55]. Consistently, Lian et al. [62] reported that SPI (and its hydrolysate) complexed with root-bark tannins via covalent and non-covalent interactions showed reduced surface -SH alongside increased intermolecular disulfide formation, in line with the trends observed. Overall, the reduction and modification of surface free-SH groups by SI weakens sulfhydryl availability, which may affect the oxidative stability and functional performance of SPI-based food systems.

### 3.12. Emulsifying Activity Index and Emulsifying Stability Index

EAI reflects the interfacial area generated per unit protein during homogenization, while ESI evaluates resistance to destabilization over time [63]. As shown in Figure 6E, SPI displayed relatively low EAI and ESI values (57.81 m^2^/g and 50.04 min, respectively). Incorporation of SI significantly increased both indices (*p* < 0.05). Notably, SPI-SI3 exhibited the highest values (72.24 m^2^/g for EAI and 93.17 min for ESI), indicating enhanced interfacial adsorption capacity and improved film stability of the SPI-SI complexes at moderate SI concentration—this behavior can be attributed to the increased H_0_ revealed by fluorescence spectroscopy, which facilitates interfacial adsorption, together with a balanced electrostatic repulsion indicated by zeta potential measurements. The improved ESI is further supported by the decrease in free-SH content, suggesting the formation of a more cross-linked and stable interfacial film. Therefore, EAI and ESI increased [64]. However, at excessive SI concentrations, EAI and ESI decreased; likely, protein aggregation and reduced conformational flexibility, as these factors limit the movement, and proteins reorganize at the boundary between oil and water. Consequently, EAI and ESI decreased. Overall, incorporation of SI optimized emulsifying properties, supporting rational design of protein–polyphenol complexes for functional emulsions.

### 3.13. Water Holding Capacity and Oil Binding Capacity

WHC and OBC are crucial functional characteristics of protein-derived ingredients, reflecting their ability to absorb and retain water or oil within a matrix under processing conditions [65]. Figure 6F shows that the WHC of SPI was 2.70%, whereas its OBC was 4.20%. Incorporating SI caused a small but meaningful enhancement in WHC (*p* < 0.05), with SPI-SI3 reaching 2.90%, likely due to enhanced hydration via hydrogen bonding between phenolic hydroxyls and water [66,67]. In contrast, OBC significantly decreased with increasing SI concentration (*p* < 0.05), suggesting that SI binding induced structural rearrangements that exposed polar residues while masking hydrophobic domains, thereby reducing available oil-binding sites [68]. Consequently, a gradual decline in OBC was observed. In general, the SPI-SI interactions enhanced H_0_, improved the WHC, and lowered the OBC, trends that were consistent with the variations noted in zeta potential and solubility. These results collectively indicate that SI association drives the system toward increased hydration capacity while diminishing lipid affinity.

## 4. Conclusions

In this research, SPI-SI complexes prepared through the pH-driven method showed significantly enhanced physicochemical, structural, and functional characteristics in comparison to SPI. SI binding promoted partial unfolding of SPI, leading to looser conformations, altered secondary and tertiary structures, and enhanced protein aggregation. These structural modifications contributed to reduced particle size, lower surface free-SH group content, and improved solubility, WHC, EAI, and ESI, with optimal performance observed at 3.0 mg/mL. Notably, the concentration-dependent effects of SI on SPI assembly were further illustrated by the dynamic trends observed in particle size, PDI, zeta potential, and H_0_. Collectively, the enhanced solubility, interfacial, and water-binding properties of SPI-SI complexes indicate their potential application as multifunctional stabilizers or emulsifiers in high-protein beverages, emulsified sauces, and plant-based dairy alternatives. Meanwhile, the binding of SI can improve the functional efficacy of SPI, offering a promising strategy for valorizing soybean meal and broadening SPI applications in functional foods. Subsequent research ought to prioritize the refinement of SI concentrations to enhance functional advantages, evaluate their use in intricate food systems (such as dairy matrices) concerning stability and antioxidant potential, and investigate their capabilities for delivering water-soluble nutrients.

## Data Availability

The original contributions presented in the study are included in the article, further inquiries can be directed to the corresponding authors.
